# More expression of BDNF associates with lung squamous cell carcinoma and is critical to the proliferation and invasion of lung cancer cells

**DOI:** 10.1186/s12885-016-2218-0

**Published:** 2016-02-29

**Authors:** Si-yang Zhang, Lin-ping Hui, Chun-yan Li, Jian Gao, Ze-shi Cui, Xue-shan Qiu

**Affiliations:** Center of Laboratory Technology and Experimental Medicine, China Medical University, No.77 Puhe Road, Shenyang North New Area, Shenyang, Liaoning People’s Republic of China; Laboratory Center, the Fourth Affiliated Hospital of China Medical University, No.4 Chongshan East Road, Huanggu District, Shenyang, Liaoning People’s Republic of China; Department of Pathology, the First Affiliated Hospital of China Medical University, No.155 Nanjing North Street, Heping District, Shenyang, Liaoning People’s Republic of China

**Keywords:** BDNF, Expression, Knockdown, Lung SCC, ADC

## Abstract

**Background:**

Brain-derived neurotrophic factor (BDNF) has been reported to promote tumorigenesis and progression in several human malignancies. The purpose of this study was to explore the function of BDNF in lung squamous cell carcinoma (SCC) and adenocarcinoma (ADC).

**Methods:**

The expression of BDNF was examined in 110 samples of lung SCC and ADC by immunohistochemistry. The protein level of BDNF was examined in 25 lung SCC or ADC samples and paired non-tumors by western blot. BDNF expression was also evaluated in human bronchial epithelial cells (HBE) and 4 lung cancer cell lines using western blot. Three BDNF mRNA variants containing exons IV, VI and IX were evaluated in HBE, two SCC (SK, LK2) and two ADC (A549, LTE) cell lines by RT-PCR. The expression and secretion of BDNF were also determined in cells using western blot and ELISA. Then the shRNA specific for BDNF was transfected into LK2 or A549 cells to further elucidate the BDNF knockdown on cell proliferation, apoptosis and invasion, which were confirmed by MTT, flow cytometry and transwell examinations.

**Results:**

71.8 % (79 out of 110) of lung SCC and ADC samples were detected positive BDNF, and high expression of BDNF was significantly correlated with histological type and T stage. Compared with non-tumorous counterparts, BDNF was apparently overexpressed in SCC and ADC tissues. In cell studies, the extensive expression and secretion of BDNF were demonstrated in lung cancer cells compared with HBE cells. Interestingly, the expressions of BDNF mRNA variant IV and VI were identical in all cells examined. However, more expression of BDNF mRNA variant IX was found in SK and LK2 cells. The apoptotic cells were increased, and the cell proliferation and invasion were both attenuated once the expression of BDNF was inhibited. When retreated by rhBDNF, BDNF knockdown cells showed less apoptotic or more proliferative and invasive.

**Conclusions:**

Our data show that BDNF probably facilitates the tumorigenesis of lung SCC and ADC. The expression of BDNF mRNA variant IX is probably more helpful to the upregulation of BDNF in SCC, and intervening the production of BDNF could be a possible strategy to lung cancer therapy.

## Background

Lung cancer is a common and serious malignant tumor worldwide, and the incidence and mortality of lung cancer are increasing every year. Lung SCC and ADC are the primary histological classification of lung cancer and included in this study. The outcome of patients with lung cancer mainly depends on tumor development and evolution, which are closely associated with the expression of tumor related genes. More research should be performed to identify the regulatory function of those genes.

Brain-derived neurotrophic factor (BDNF) is a member of the neurotrophin family. BDNF plays an important role in the development and regeneration of the neurons. Binding of BDNF to its major receptor, tropomyosin-related receptor kinase B (TrkB) with high affinity and specificity [1], leads to the activations of various downstream signalings, including PI3K/AKT, RAS/ERK, PLC/PKC, AMPK/ACC and JAK/STAT pathways [2]. BDNF and TrkB have been reported to promote tumorigenesis and progression in several human malignancies such as neuroblastoma [3], breast [4], lung [5], prostate [6], and colon cancer [7]. Studies have shown that BDNF/TrkB signaling was involved in proliferative [8] or invasive properties [9]. These findings indicated that BDNF/TrkB signaling was closely associated with tumor progression [10], and it has emerged as a potential therapeutic target [11].

Researches focused on the function of BDNF/TrkB in lung cancer were also performed in recent years. Ricci A and his colleagues recognized the importance of neurotrophins and receptors family for human lung cancer [12]. It has been reported that the expression of TrkB and BDNF was associated with poor prognosis in non-small cell lung cancer [13] and TrkB/BDNF signaling pathway could be a therapeutic target for pulmonary large cell neuroendocrine carcinoma [14]. We previously demonstrated the involvement of the BDNF/TrkB pathway in the invasion of A549 cells [15].

However, despite the considerable amount of studies on BDNF have been reported in human cancer, here we investigated the function of BDNF in lung SCC and ADC. We reported here that the overexpression of BDNF was common in SCC and ADC, particularly correlated with histological type and T stage. We also reported that the expression and secretion of BDNF were more extensive in lung cancer cells. Moreover, the BDNF mRNA variant containing exon IX was important to the higher expression in SCC cells, and BDNF was critical to the proliferation and invasion of lung cancer cells.

## Methods

### Lung cancer samples

110 cases of lung SCC and ADC samples were all from the Pathology Department in China Medical University. The ethical approval was given by the Medical Research Ethics Committee of China Medical University. All participants were provided the written informed consent before enrolment in this study and the agreements were obtained. The enrolled cases did not receive any chemotherapy or radiation therapy before curative surgical resection. Formalin-fixed paraffin-embedded sections of tumor were stained with hematoxylin and eosin (HE), and diagnosed according to the guidelines of classification of lung and pleural tumors (2004) and the TNM staging system (1997) of WHO by two senior pathologists. The histological type, differentiation, stage and lymph node metastasis were determined accordingly and summarized in Table 1.

**Table 1:** Statistical analysis on the correlations between BDNF expression by immunohistochemical staining in 110 lung cancer samples and the clinicopathological parameters

Clinicopathological Characteristics	Cases (*n* = 110)	Low expression (*n* = 49)	High expression (*n* = 61)	*p*-value
Histological type				
Squamous cell carcinoma	53	20	33	0.017
Adenocarcinoma	57	29	18	
Grade				
well-moderate	76	31	45	0.236
poor	34	18	16	
Stage				
I-II	72	36	36	0.113
III	38	13	25	
Tumor size and extension				
T1-T2	75	39	36	0.021
T3	35	10	25	
Lymph node metastasis				
No	68	30	38	0.532
Yes	42	19	23	

### Immunohistochemistry

110 paraffin sections of lung SCC and ADC were deparaffinized and rehydrated routinely. The sections were heated in 0.1 mol/L Tris–HCl at pH10 in an autoclave sterilizer for 1 min for the recovery of antigen. The sections were incubated with 3 % H_2_O_2_ to eliminate endogenous peroxidase, followed by 5 % goat serum to avoid unspecific binding of antibody at 37 °C for 1 h. The sections were then incubated with primary mouse monoclonal antibody specific for BDNF (1:100 dilution, Abcam) overnight at 4 °C. The next day, sections were incubated with goat anti-mouse IgG and streptavidin-peroxidase (SP) complex at 37 °C for 40 min (SP kit, Maxim, China), and then developed with 3,3′-diaminobenzidine (DAB). Neuroblastoma was used as positive control for BDNF, and nonimmune goat IgG instead of BDNF antibody was used as negative control. Two senior pathologists assessed the immunostained sections separately. The distinguishing brown particles in cytoplasm were regarded as BDNF-positive. The intensity of BDNF immunostaining (0 = negative, 1 = weak, 2 = intense) and the percentage of positive tumor cells (≤50 % = 1, >50 % = 2) were evaluated in at least 5 high power fields (×400 magnification). The scores of each tumor section were multiplied to generate a final score of 0, 1, 2 or 4, and the samples were finally defined as high expression: score >2; or low expression: score ≤2 (including negative: score 0).

### Cells culture and transfection

Human bronchial epithelial cells (HBE), lung SCC cells (SK-MES-1, LK2) and ADC cells (A549, LTE) were cryopreserved in our department. HBE, A549 and LTE cells were cultured in RPMI1640 (Gibco, USA), and SK, LK2 cells were cultured using DMEM (Gibco, USA), supplemented with 10 % fetal bovine serum (FBS), in an incubator at 37 °C with 5 % CO_2_. LK2 and A549 cells were selected to perform the establishment of stable cell strain with BDNF knockdown for their better condition and vitality. They (50-60 % confluence) were transfected with either BDNF-shRNA or scrambled control (Genechem, China) using Lipofectamin 2000 (Invitrogen, USA), according to the manufacturer’s guidelines. The qualified cell clones were screened by G418, and the decreased expression of BDNF was confirmed by western blot. When indicated, those transfected cells were retreated with recombinant human BDNF (rhBDNF, 100 ng/mL, Peprotech, USA). Cells were used for proteins extraction or cell biological assays as described below. The experiments for cells were performed in triplicates.

### Western blot

25 cases of fresh SCC (10) and ADC (15) were obtained from the Pathology Department. Tissues or cells were washed with ice-cold phosphate buffer saline (PBS) and lysed in RIPA lysis buffer containing protease inhibitor (Roche, USA). The homogenate was centrifuged at 15,000 g at 4 °C for 30 min. The supernatant was collected and protein content was quantified by the method of bicinchoninic acid (BCA) assay (Pierce). 60 μg of protein sample was loaded in sodium dodecyl sulfate-polyacrylamide gel electrophoresis (SDS-PAGE) and transferred to polyvinylidene fluoride (PVDF) membrane. 5 % bovine serum albumin (BSA) was used to block unspecific binding of antibodies to membrane. Then the primary antibodies containing mouse monoclonal anti-BDNF (1:200, Abcam, USA) or rabbit polyclonal anti-BDNF (1:200, Sangon, China) and anti-β-actin (1:1000, Santa Cruz, USA) were incubated with membranes at 4 °C over night. The membranes were then incubated with goat anti-mouse or rabbit IgG (1:2000, ZhongShan, China) at 37 °C for 2 h. The specific protein bands were visualized using the enhanced chemiluminescence (ECL) method (TranGen Biotech, China), using the DNR Imaging System. The Image-Pro Plus 6.0 software was used to perform semi-quantitative analysis of the optical density of each band. The ratio between the optical density of BDNF and β-actin of the same sample was used to indicate the relative level of BDNF expression.

### ELISA

Human BDNF Quantikine ELISA kit (R&D Systems, Minneapolis, MN, USA) was used to measure the protein concentration of BDNF in the supernatant collected by centrifugation from cultured cells for 24 or 48 h. BDNF secretion was evaluated according to the manufacturer’s instructions. In general, 50 μL of sample or standard was mixed with 100 μL assay diluent and added to the microplate wells, incubating at room temperature for 2 h. The mixture was incubated at room temperature for another 1 h after adding 100 μL of BDNF conjugate. The development reaction was performed with 200 μL of substrate solution after several washes. Then the absorbance value was shown by a microplate reader at 450 nm, with a calibration wavelength of 570 nm.

### Reverse transcription polymerase chain reaction

Total RNA of cells was extracted using Trizol (Invitrogen, USA). cDNA was produced by reverse transcription reaction of 5 μg total RNA using GoScript Reverse Transcription System (Promega, USA). The primer sequences used in this study were listed in Table 2. The lengths of the PCR products using the reverse primer hBDNF_IXbAS in combination with the following forward primers were hBDNF_IVS, 412 bp; hBDNF_VIS, 494, 387, and 369 bp; and hBDNF_IXS, 597 and 363 bp, respectively (as described by Prof. Pruunsild) [16]. PCR conditions were set as follows: initial denaturation at 94 °C for 2 min; 35 cycles of denaturation at 94 °C for 30s; annealing at appropriate temperature listed in the Table 1 for 30s; elongation at 72 °C for 30s; final elongation at 72 °C for 5 min. The PCR conditions for β-actin were similar to that for BDNF mRNA variants, except for annealing at 55 °C and 30 cycles. The specific bands were visualized using the ChemiImager 5500 (Alpha Innotech, USA), and the Image-Pro Plus 6.0 software was used for the semi-quantitative analysis of the optical density of each band.

**Table 2:** The primers of 3 BDNF mRNA variants and β-actin used in this study

mRNA		Sequences	Annealing temperature
hBDNF_IVS	forward	5′-GCTGCAGAACAGAAGGAGTACA-3′	61.8 °C
	reverse	5′-GTCCTCATCCAACAGCTCTTCTATC-3′	
hBDNF_VIS	forward	5′-GGCTTTAATGAGACACCCACCGC-3′	63.3 °C
	reverse	5′-GTCCTCATCCAACAGCTCTTCTATC-3′	
hBDNF_IXS	forward	5′-TTTCTCGTGACAGCATGAGCAG-3′	60.6 °C
	reverse	5′-GTCCTCATCCAACAGCTCTTCTATC-3′	
β-actin	forward	5′-TGAGTCTCCTTTGGAACTCTGC-3′	55 °C
	reverse	5′-TAGCAACGTACATGGCTGGG-3′	

### CCK-8 assay

The cell proliferation was evaluated using CCK-8 (DOJINDO, Japan) assay, which was lasted for 5 days. Cells were digested, counted and adjusted to 1 × 10^3^/100 μL, and seeded in five 96-well plates simultaneously. Every day, a random plate was selected for the measurements of absorbance, and cells in other plates were cultured for the rest of days. The absorbance of each well was measured in a microplate reader (SpectraMax Plus 384, USA) at 450 nm at 4 h after 10 μL CCK-8 was added. The average absorbance (Y-axis) and time (X-axis) were applied to draw the cell growth curves. Data indicated are mean value of three individual wells.

### Cell apoptosis assay

The Annexin V-FITC (fluorescein isothiocyanate) apoptosis detection kit (BD Biosciences, USA) was used to monitor cell apoptosis by flow cytometry, according to the manufacturer’s protocol. Cells were rinsed twice using ice-cold PBS and resuspended in 1 × binding buffer (1 × 10^6^/mL). 100 μL (1 × 10^5^) cells were gently mixed with 5 μL PI and 5 μL Annexin V-FITC, and incubated for 15 min at room temperature in dark. Cells were supplemented with another 400 μL 1 × binding buffer, and examined in a flow cytometer. Data are representative of three individual tests.

### Cell invasion analysis

The 24-well Transwell chamber (Costar, USA) was used to perform cell invasion assay. Cells were digested, counted and adjusted to 1 × 10^4^, and seeded in the upper chamber with an 8 μm pore size insert in a 24-well plate. The inserts were precoated with Matrigel (1:6 dilution, BD Biosciences) and cells were cultured in medium containing 1 % FBS. They were driven to invade Matrigel and migrate towards medium containing rhBDNF (100 ng/mL) or 10 % FBS in the bottom chamber for 24 h. After removing the detained cells on the upper surface of membrane with a cotton tip, the migratory cells on the under surface were fixed using 4 % paraformaldehyde and stained with hematoxylin. The number of invaded cells was counted in 5 randomly selected fields under a microscope of 200× power. Data expressed are mean value of three individual wells.

### Statistical analysis

SPSS 13.0 statistical analysis software was used to perform data statistics. *χ*2-test was applied to evaluate the correlations of BDNF expression and clinicopathological parameters for the immunohistochemical results. The *t*-test was used to evaluate the difference of BDNF expression between tumors and non-tumor counterparts. One-way ANOVA was used to evaluate the differences between variously treated cells. All data were shown as mean ± SD and results were regarded statistically significant when the *p* value <0.05.

## Results

### BDNF expression in specimens of lung SCC and ADC by Immunohistochemistry

**Fig. 1:**
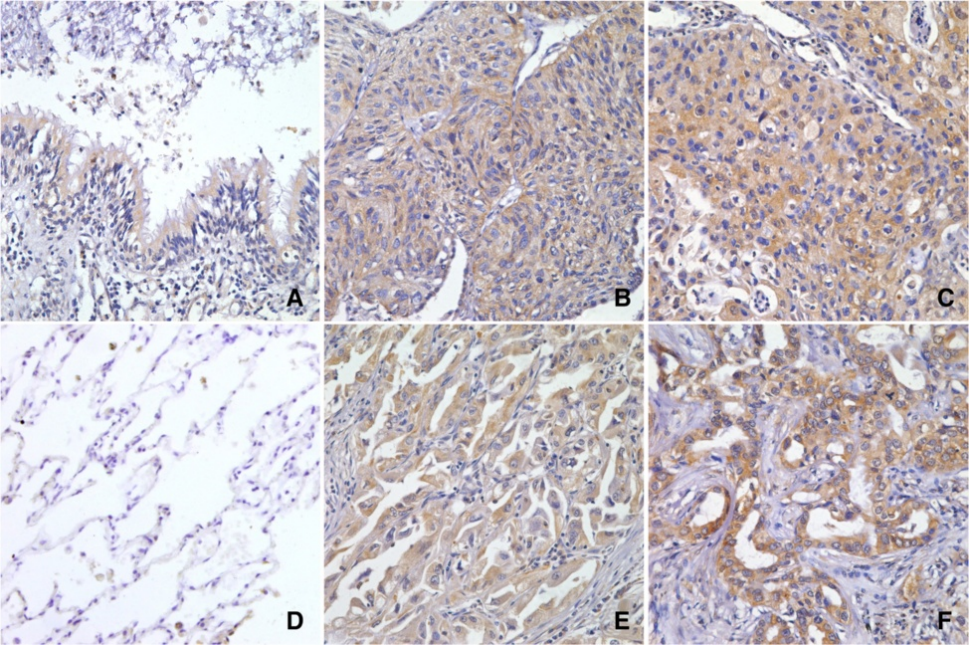
BDNF expression in bronchial and alveolar epithelium, SCC and ADC tissues by immunohistochemical staining. Hematoxylin was counterstained for nuclei. Weak expression of BDNF was shown in bronchial epithelial cells (**a**), and no expression was found in alveolar epithelium (**d**). SCC showed positive expression of BDNF (**b** and **c**), including the moderate staining of T1 stage (B), and intense staining of T3 stage (**c**). ADC showed positive expression of BDNF (**e** and **f**), including the moderate staining of T1 stage (**e**), and intense staining of T3 stage (**f**). (magnification, 400×)

Weak expression of BDNF was shown in the cytoplasm of bronchial epithelial cells (Fig. 1a), and no expression was found in alveolar epithelium (Fig. 1d). BDNF immunostaining was observed in the cytoplasm of cancer cells. Positive BDNF was found in 79 (71.8 %) neoplastic sections. We considered that 61 (55.5 %) cases were high expression (scores >2) and 49 cases (44.5 %) were low expression (scores ≤2), as elaborated in Methods. BDNF was reported to be correlated with tumor growth, invasiveness and metastasis, so the association between BDNF expression and clinicopathological characteristics was analyzed statistically, as shown in Table 1. BDNF immunostaining was stronger in tumors of SCC (vs. ADC, *p* = 0.017) and T3 (vs. T1-T2, *p* = 0.021). And no significant difference of BDNF expression was found between tumors with various differentiation (well-moderate vs poor, *p* = 0.236), stage I-II (vs. III, *p* = 0.113) and lymph node status (metastasis vs no metastasis, *p* = 0.532).

### BDNF expression in 25 cases of tumor and paired non-tumor by western blot

**Fig. 2:**
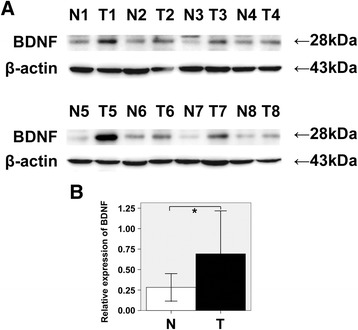
**a** Expression of BDNF was detected by western blot in paired tumors (T) and non-tumors (N) from 8 of 25 lung cancer patients, and 4 of which were SCC (T1, T3, T5, T7), the other 4 were ADC (T2, T4, T6, T8). It was shown that BDNF expression was up-regulated in tumor compared with non-tumor of the same patient. β-actin was used as a reference control to ensure the equal protein quantity in all lanes. **b** The ratio between the optical density of BDNF and β-actin of the same sample was calculated and plotted. The significant difference of BDNF between tumors (T) and non-tumors (N) were analyzed statistically. BDNF immunoreactivity is greater in neoplastic tissues

Western blot analysis was used to detect BDNF expression in 10 SCC and 15 ADC cases of lung cancer and non-tumorous tissue distant from the primary tumor of the same case. The overexpression of BDNF was found in 20 tumor samples in comparison with the non-tumor counterparts (*p* = 0.000). The specific bands for BDNF of eight samples are shown in Fig. 2a, and the relative optical density of the tumor (T) and non-tumor (N) tissues of the same patient was measured and expressed graphically (Fig. 2b).

### BDNF expression in HBE and four lung cancer cell lines by western blot

**Fig. 3:**
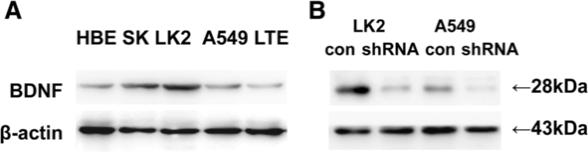
**a** BDNF expression in HBE cells, two SCC cells (SK and LK2) and two ADC cells (A549 and LTE) by western blot analysis. The ratio between the optical density of BDNF and β-actin of the same cells was used to indicate the relative level of BDNF expression. The expression of BDNF was rare in HBE cells, and that was much higher in other 4 lung cancer cells. **b** The decreased expression of BDNF by specific shRNA was shown in LK2 or A549 cells. β-actin was used as a reference control to ensure the equal protein quantity in all lanes. The experiments for each cell line were completed in triplicates

The expression of BDNF was also examined in HBE, two lung SCC (SK, LK2) and two ADC (A549, LTE) cell lines using western blot. We showed that the expression of BDNF represented by the specific bands of 28 kDa was almost undetectable in HBE cells. However, the IOD indicative of BDNF protein level was excessively high in lung cancer cells (Fig. 3). Statistical analysis confirmed that SK and LK2 cells expressed more BDNF than A549 and LTE cells (*p* = 0.000).

### The secretory BDNF in supernatants of cells by ELISA

BDNF is a secretory cytokine, which is prepared by tumor cells and promotes growth and survival of themselves [17]. In the present study, BDNF secreted by HBE, SK, LK2, A549 and LTE cells was measured by ELISA assays. Our results showed that BDNF in supernatant of HBE and A549 cells at 24 or 48 h were not detected. BDNF in supernatant of LTE cells was undetected at 24 h, which was 30.5 ± 18.0 pg/mL at 48 h. The concentration of BDNF in supernatant of SK and LK2 cells were 38.3 ± 15.5 and 29.0 ± 11.6 pg/mL at 24 h, and 150.5 ± 54.0 pg/mL and 212.0 ± 88.0 pg/mL at 48 h, respectively. These results indicated that more BDNF was produced by SK and LK2 cells.

### The expression of BDNF mRNA variants in cells by RT-PCR

**Fig. 4:**
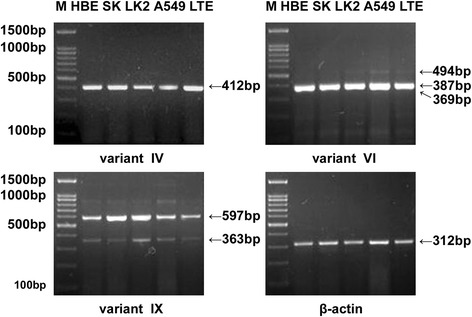
The expression of BDNF mRNA variants respectively containing exons IV, VI and IX in HBE, SK, LK2, A549 and LTE cells by RT-PCR. BDNF mRNA levels remained invariable in variants containing exons IV and VI. SK and LK2 cells showed much higher level of BDNF mRNA variant containing exon IX, compared with HBE, A549 and LTE cells. β-actin was used as a loading control to assure equal amounts of protein in all lanes. The experiments for each cell line were repeated at least three times

BDNF has multiple alternative splicing variants and plays diverse biological functions in mammals, including neuronal survival, cell differentiation and tumor development. The multiple BDNF alternative splicing variants are formed to achieve precise temporal and spatial expression of functional BDNF. The expressions of BDNF mRNA variants containing exons IV, VI and IX were selected according to previous reports [16], and investigated in this study. The mRNA levels were found identical in variants containing exons IV and VI in cells examined. However, the mRNA level was different in variants containing the exon IX (Fig. 4). Statistical analysis proved that SK and LK2 cells expressed more BDNF variant IX than HBE, A549 and LTE cells (*p* = 0.000).

### Supppression of cell proliferation by BDNF-shRNA

**Fig. 5:**
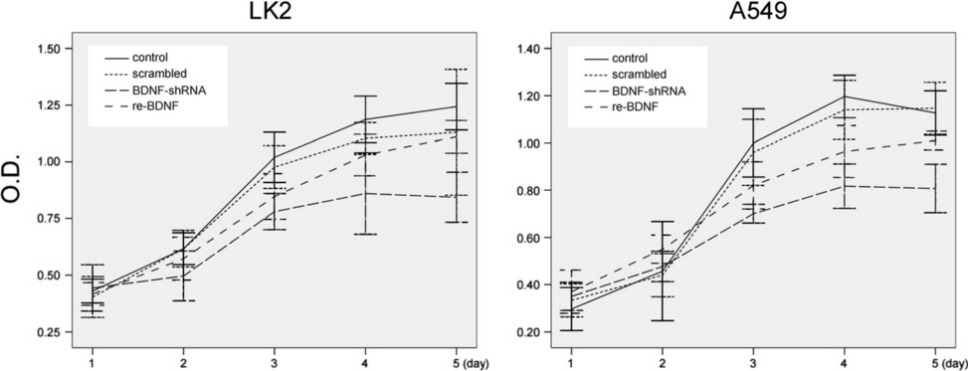
The effects of BDNF knockdown or retreatment on cell proliferation. Cell growth curves showed that both LK2 (left panel) and A549 (right panel) cells with BDNF-shRNA transfection presented a decreased proliferative activity compared with other groups during the 5 days. Furthermore, both cells proliferation was facilitated after rhBDNF retreatment. Data are indicated as mean ± SD of three replicates

LK2 and A549 cells were transfected with specific shRNA to explore the function of BDNF on cell proliferation, apoptosis and invasion. Fig. 3b confirmed the decreased expression of BDNF in transfected cells. We found that 2 days after LK2 were seeded, and 3 days after A549 cells were seeded, the absorbance in BDNF-shRNA, scrambled and control groups was statistically different (*p* = 0.025 and *p* = 0.002). According to the cell growth curves during the 5 days, we found that both LK2 and A549 cells with BDNF-shRNA transfection showed a decreased proliferative activity compared with other groups. When retreated with rhBDNF, BDNF knockdown LK2 or A549 cells showed enhanced proliferative abilities (Fig. 5). These results showed that suppression of BDNF expression interfered with the proliferation of LK2 and A549 cells.

### More apoptotic cells with BDNF-knockdown

**Fig. 6:**
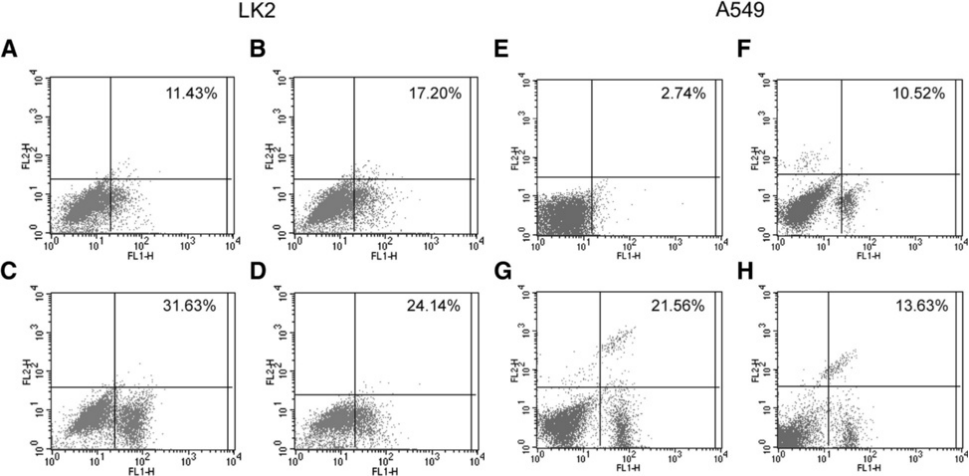
The effects of BDNF knockdown or retreatment on cell apoptosis. The apoptotic rate of BDNF knockdown cells was evidently increased or decreased with rhBDNF treatment. **a** LK2 cells untreated. **b** scrambled shRNA transfected LK2 cells. **c** LK2 cells of BDNF knockdown. **d** LK2 cells with rhBDNF retreatment. **e** A549 cells untreated. **f** scrambled shRNA transfected A549 cells. **g** A549 cells of BDNF knockdown. **h** A549 cells with rhBDNF retreatment. The data are shown as mean ± SD of three individual experiments

Cell apoptosis was examined in this study to investigate whether BDNF facilitated overgrowth of cells by apoptosis inhibition. The apoptotic rates of LK2 cells in BDNF-shRNA, scrambled and control groups were 30.8 ± 1.9 %, 16.3 ± 2.7 % and 9.8 ± 1.6 % (*p* = 0.000; Fig. 6a, b, c). The apoptotic rates of A549 cells in the above groups were 21.7 ± 2.3 %, 9.1 ± 1.5 % and 4.1 ± 1.3 % (*p* = 0.000; Fig. 6e, f, g). When retreated with rhBDNF, the apoptotic LK2 or A549 cells with BDNF knockdown were apparently reduced (Fig. 6d and h). Our results showed that apoptosis was significantly induced in BDNF shRNA-transfected cells.

### Down-regulation of BDNF on the invasive potential of shRNA-transfected cells

**Fig. 7:**
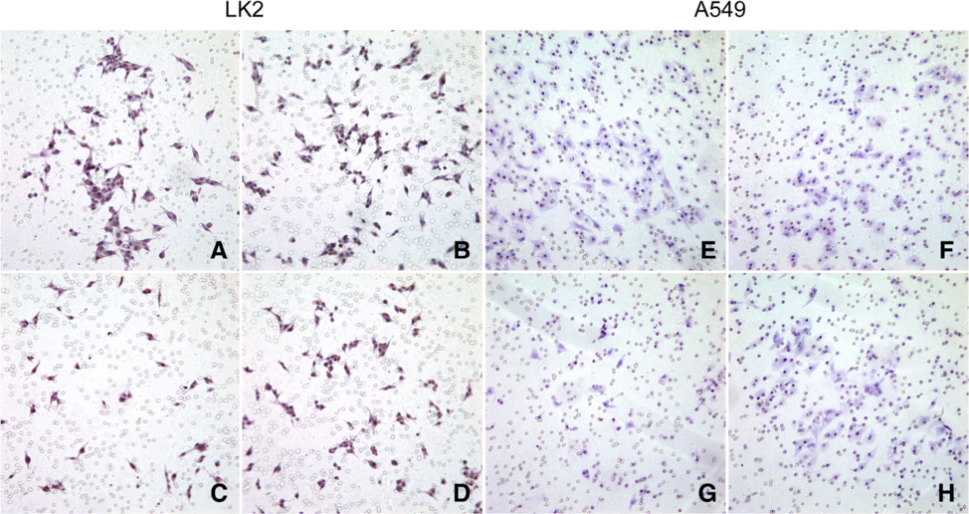
The effects of BDNF knockdown or retreatment on cell invasion. **a** LK2 cells untreated. **b** scrambled shRNA transfected LK2 cells. **c** cell invasion was inhibited in BDNF-shRNA transfected LK2 cells. **d** more invasive cells were shown in rhBDNF treated BDNF-knockdown LK2 cells. **e** A549 cells untreated. **f** scrambled shRNA transfected A549 cells. **g** the number of invasive A549 cells with BDNF-shRNA transfection was reduced. **h** cells were more invasive after rhBDNF treatment. The values are mean ± SD of three independent experiments

Since BDNF overexpression was common in more aggressive tumours, the stable cells of LK2 or A549 with low expression of BDNF were used to determine its contribution to cell invasion. The numbers of invasive LK2 or A549 cells in BDNF-shRNA or scrambled shRNA transfected and control groups were 31.3 ± 4.2, 72.0 ± 4.4, 65.7 ± 7.0 and 29.7 ± 4.2, 63.0 ± 7.2, 66.0 ± 7.2, respectively (*p* = 0.000; Fig. 7a-c and e-g). However, in the group of LK2 or A549 cells retreated with rhBDNF, chemotaxis of rhBDNF was shown as 45.7 ± 4.7 or 47.0 ± 5.6, which was both more than BDNF-shRNA group (*p* = 0.000; Fig. 7d and h). These results showed that BDNF knockdown significantly attenuated the invasive ability of transfected cells.

## Discussion

Brain-derived neurotrophic factor, also known as BDNF, is a member of the “neurotrophin” family of growth factors, helping to support the survival of existing neurons, and encourage the growth and differentiation of new neurons and synapses [18]. The up-regulation of BDNF was found in a variety of primary human tumors, including breast cancer [19], hepatocellular carcinoma [20], and bladder cancer [21], suggesting a significant role of BDNF in the development and progression of cancer. The primary receptor for BDNF, TrkB is critical in lung cancer development. A recent study by Sinkevicius KW found that the receptor TrkB deficiency significantly reduced metastasis of a lung adenocarcinoma model [22]. Götz R also revealed that the cooperation of TrkB and EGFR signaling enhances migration and dispersal of lung tumor cells [23].

In this study, we evaluated the expression of BDNF in lung SCC and ADC. We found a statistical evidence of BDNF up-regulation in lung cancer, compared with their non-tumor counterparts. BDNF overexpression was found extensively in most of neoplastic tissues, supporting its potential role in lung tumorigenesis. We also examined 110 FFPE sections of lung SCC and ADC by means of immunohistochemistry to reveal the clinical significance of BDNF for lung cancer. We found that tumors with high BDNF expression had an advanced T stage, and SCC expressed more BDNF. It has been reported that the positive TrkB staining in SCC correlated specifically with improved disease-specific survival and overall survival [24]. Furthermore, the lineage transition in SCC and ADC of lung cancer was presumed for the clinical observation of mixed components in adenosquamous carcinoma recently. Studies have shown the evidence for the plasticity of lung cancer, that the transdifferentiation from SCC to ADC under implicated circumstances [25, 26]. However, the significance of BDNF in lung SCC still required intensive research. These results suggested that BDNF was critical in the tumorigenesis of lung cancer. Further studies and more samples are required to investigate the relationship between BDNF and TrkB in lung SCC and ADC.

It has been reported that BDNF is a secretory protein, produced by tumor cells to promote their growth and survival [27]. Therefore, BDNF in culture supernatant was assessed in HBE and four lung cancer cell lines. As we expected, the SCC cells of LK2 and SK had more secretion of BDNF in supernatant, compared to HBE and the ADC cells of A549 and LTE. We also examined the expression of BDNF by western blot in HBE and other lung cancer cells. We showed that BDNF expression represented by the specific protein band of 28 kDa was quite weak in HBE cells, while SK and LK2 cells expressed higher level of BDNF, compared with A549 and LTE cells. However, besides the specific BDNF bands, various protein bands were also found in cells, which were not present in tissue samples. We presumed that those unexpected bands were proteins probably containing its precursor proBDNF [28], or some unknown BDNF protein subtypes came from spliced mRNA variants, which requires further identifying and recognizing investigations.

It has been reported that 17 splice variants when BDNF gene was transcripted into mRNAs, and each variant is expressed in specific tissues and cells. Studies have shown that human BDNF alternative transcripts are expressed in a tissue-specific manner. According to the previous research, three BDNF mRNA transcripts containing exons IV, VI or IX, which are expressed relatively higher in lung tissue, were selected to be examined in this study to evaluate the significance of the above variants to the higher expression of BDNF in lung cancer cells. We found that the mRNA level of variants IV and VI was identical, only the mRNA level of variant IX was different among cells examined. SCC cells of SK and LK2 with more expression of BDNF protein had higher level of the mRNA variant IX. These results suggested that variant IX was responsible for the higher expression and secretion of BDNF protein, which was probably helpful to the development of SCC.

We have previously supported that blocking of receptor TrkB signaling inhibited invasion of A549 cells. In this study, we explored the function of BDNF on proliferation, apoptosis and invasion by specific shRNA transfection in LK2 and A549 cells. It was confirmed that blocking of BDNF expression inhibited cell proliferation and invasion, and promoted cell apoptosis. Our data indicate that BDNF promotes cell proliferation and invasion, and silencing BDNF probably confers the disadvantage to the growth of lung cancer cells.

We have previously confirmed that TrkB was overexpressed in lung cancer, BDNF facilitated A549 cell invasion by inducing phosphorylation of Pyk2-tyr402, which is a newly found signaling associated with invasion of lung cancer cells. Pyk2 is also called Ca^2+^-dependent tyrosine kinase, which is involved in the regulation of Ca^2+^ flow-mediated signaling activations and cell migration. Therefore, the Ca^2+^/Pyk2 signaling involved in cell invasion were investigated recently. The LK2 and A549 cells with shBDNF transfection were stimulated by recombined human BDNF. We observed the elevated Ca^2+^ concentration and Pyk2-tyr402 phosphrylation, and cells invasion was also enhanced. Ca^2+^ concentration was significantly elevated after BDNF stimulation, and that was much lower in cells pretreated with endoplasmic reticulum Ca^2+^ channel protein IP3R blocker 2-APB than control cells, which indicated that BDNF promoted Ca^2+^ release from endoplasmic reticulum through IP3R. We are proposing to focus on Ca^2+^ signaling associated with invasion of lung cancer cells in our future studies.

## Conclusion

In conclusion, our study demonstrated that overexpression of BDNF was evident in lung SCC and ADC, and the increased expression of BDNF protein in SCC cells was closely correlated with higher level of its spliced mRNA variant containing exon IX. Knockdown of BDNF using RNA interfering showed the decreased activity of proliferation and invasion in LK2 and A549 cells, which indicated that BDNF may play a decisive role in tumorigenesis of lung SCC and ADC, whose growth and survival were probably facilitated by BDNF secreted by themselves. The suppression of BDNF may provide a significant target for inhibitory strategies for the development of lung cancer, which requires further investigations.

## Abbreviations

ADC: adenocarcinoma
AMPK/ACC: Adenosine 5′-monophosphate (AMP)-activated protein kinase/acetyl-coA carboxylase
BCA: bicinchoninic acid
BDNF: brain-derived neurotrophic factor
BSA: bovine serum albumin
CCK-8: cell counting kit
DAB: 3,3′-diaminobenzidine
ECL: enhanced chemiluminescence
ELISA: enzyme linked immunosorbent assay
ERK: extracellular signal-regulated kinase
FBS: fetal bovine serum
FFPE: formalin-fixed paraffin-embedded
FITC: fluorescein isothiocyanate
HBE: human bronchial epithelial cell
HE: hematoxylin and eosin
IOD: integral optical density
IP3R: inositol 1,4, 5-trisphosphate receptor
JAK/STAT: Janus kinase/signal transducer and activator of transcription.
MTT: 3-(4,5-dimethyl-2-thiazolyl)-2,5-diphenyl-2-H- tetrazolium bromide
PBS: phosphate buffer saline
PI3K/AKT: phosphatidylinositol 3 kinase/protein kinase B
PLC/PKC: phospholipase C/protein kinase C
PVDF: polyvinylidene fluoride
Pyk2: proline-rich tyrosine kinase 2
rhBDNF: recombinant human BDNF
RIPA: radio-immunoprecipitation assay
SCC: squamous cell carcinoma
SD: standard deviation
SDS-PAGE: sodium dodecyl sulfate-polyacrylamide gel electrophoresis
SP: streptavidin-peroxidase
SPSS: Statistical Product and Service Solutions
TrkB: tropomyosin-related receptor kinase B
